# Buffer Solutions of Potassium Dihydrogen Phosphate and Sodium Succinate at 25 °C

**DOI:** 10.6028/jres.067A.055

**Published:** 1963-12-01

**Authors:** Maya Paabo, Roger G. Bates, Robert A. Robinson

## Abstract

A buffer mixture consisting of equal molalities (*m*) of potassium dihydrogen phosphate and sodium succinate is proposed as a useful reference point in the study of acid-base equilibria, bridging the present gap between *p*H 5.5 and *p*H 6.8. The *p*(a_H_*γ*_Cl_) at 25 °C has been determined by electromotive-force measurements for five buffer solutions in which *m* varied from 0.005 to 0.025. The conventional *pa*_H_ of each solution has been derived and found to be in good agreement with that calculated from existing data for the two equilibria concerned. The *pa*_H_ varies from 6.251 at *m*=0.005 to 6.109 at *m*= 0.025. The buffer mixture has been used successfully for the determination of the dissociation constants of 2-nitro-4-chlorophenol and 2,6-dichlorophenol.

## 1. Introduction

The useful application of the acidity function −log (*a*_H_*γ*_Cl_) or *p*(*a*_H_*γ*_Cl_) in the accurate determination of dissociation constants or *pK* values has been described [1,2].^1^ In the determination of the *pK* of a weak acid by the spectrophotometric method, a buffer solution is needed with a value of *p*(*a*_H_*γ*_Cl_) not too far removed from the *pK* of the acid. For example, in the determination of the dissociation constant of *p*-nitrophenol at 25 °C [3], *pK*=7.156, a series of equimolar mixtures of potassium dihydrogen phosphate and disodium hydrogen phosphate of different total ionic strengths were used; their *p*(*a*_H_*γ*_Cl_) values lay between 6.912 and 7.080. For 2-chloro-4-nitrophenol, *pK*=5.45, solutions equimolal with respect to both sodium hydrogen succinate and sodium succinate were used [4]; their *p*(*a*_H_*γ*_Cl_) values lay between 5.477 and 5.553.

Neither of these buffer systems would be particularly suitable for an acid with *pK* about 6.2 and we have, therefore, now studied the buffer system consisting of sodium succinate and potassium dihydrogen phosphate at equal molalities (*m*). The *p*(*a*_H_*γ*_Cl_) values of five solutions in which *m* varied from 0.005 to 0.025 have been determined at 25 °C. By the application of a conventional definition of the activity coefficient of chloride ion, conventional *pa*_H_ values of these solutions have been calculated.

## 2. Method

The electromotive-force method by which *p*(*a*_H_*γ*_Cl_) was determined and values of *pa*_H_ assigned has been thoroughly described in earlier publications [2, 5]. It consisted of the following three steps:

1. Measurement of the emf of the following cell:
  $$\text{Pt};H_{2}\left(g,1\;\text{atm}\right),\;{\text{KH}}_{2}{\text{PO}}_{4}\left(m\right),\;{\text{Na}}_{2}C_{4}H_{4}O_{4}\left(m\right),\;\text{KCl}(m^{\prime }),\;\text{AgCl};\text{Ag}$$
  at 25 °C. Five different buffer mixtures consisting of equimolal amounts of potassium di hydrogen phosphate and sodium succinate (*m* varying from 0.005 to 0.025) were studied. Measurements were made with four concentrations (*m*′) of potassium chloride, namely 0.005, 0.01, 0.015, and 0.02 molal, at each of the five buffer concentrations.
  The emf was corrected to a partial pressure of 1 atm hydrogen in the usual way, and *p*(*a*_H_*γ*_Cl_) was calculated from each value of the emf (*E*) by the formula
  $$p(a_{H}\gamma_{\text{Cl}})=\frac{(E-E{}^{\circ})F}{RT\;\ln\;10}+\log\;m^{\prime }$$ (1)
  The standard potential (*E*°) was taken to be 0.22234 v at 25 °C [6], and the coefficient *F*/(*RT* ln 10) has the value 16.9047 v^−1^ at this temperature.
2. The limiting value of *p*(*a*_H_*γ*_Cl_) in the absence of potassium chloride, termed *p*(*a*_H_*γ*_Cl_)°, was determined for each of the five buffer solutions by plotting *p*(*a*_H_*γ*_Cl_) as a function of the molality of added potassium chloride and extrapolating to 0.
3. The conventional *pa*_H_ was computed from *p*(*a*_H_*γ*_Cl_)*°* by the relationship.
  $$pa_{H}=p\left(a_{H}\gamma_{\text{Cl}}\right){}^{\circ}+\log\;\gamma_{\text{Cl}}$$ (2)
  with the use of the convention [7]
  $$\log\;\gamma_{\text{Cl}}=\frac{-A\sqrt{I}}{1+1.5\sqrt{I}}$$ (3)
  where *I* is the ionic strength and *A*, the Debye-Hückel slope, has the value 0.5108 mole ^−1/2^kg^1/2^ at 25 °C.

## 3. Experimental Procedure

The potassium dihydrogen phosphate was NBS Standard Sample 186Ib. Two different samples of sodium succinate hexahydrate, reagent grade, were used. In addition, a portion of one of the samples was recrystallized and dried to remove its water of hydration. There were no apparent differences among buffers made from the three samples of sodium succinate. Potassium chloride was recrystallized once from water. The conductivity of the distilled water used to prepare the solutions was no greater than 0.8 ×10^−6^ ohm^−1^ cm^−1^.

An increase of emf with time, the cause of which has not been determined, was observed. This increase was more rapid with the dilute solutions than with the more concentrated ones. It was therefore found necessary to make the emf measurements on the three most dilute buffer mixtures on the same day the solutions were prepared. However, the same values of the emf were obtained for fresh 0.02 *m* and 0.025 *m* buffer solutions on the day of preparation and one day later.

In terms of *pa*_H_ units, the 0.025 *m* buffer solution increased in *pa*_H_ by only 0.002 after standing two days and 0.012 after 12 days. However, the 0.01 *m* solution increased by 0.012 unit in 24 hr and the 0.005 *m* solution by 0.028 in the same time.

The stability of the primary and secondary phosphates in aqueous solution seems beyond question. Furthermore, no instability of buffer solutions composed of primary and secondary succinates was found in earlier work [8]. Nevertheless, it is advisable, for the greatest accuracy, to use the mixed buffers on the day of their preparation. This procedure was successful in the determination of the dissociation constants of 2-nitro-4-chlorophenol and 2,6-dichlorophenol (see below).

## 4. Results and Discussion

**Figure 1:**
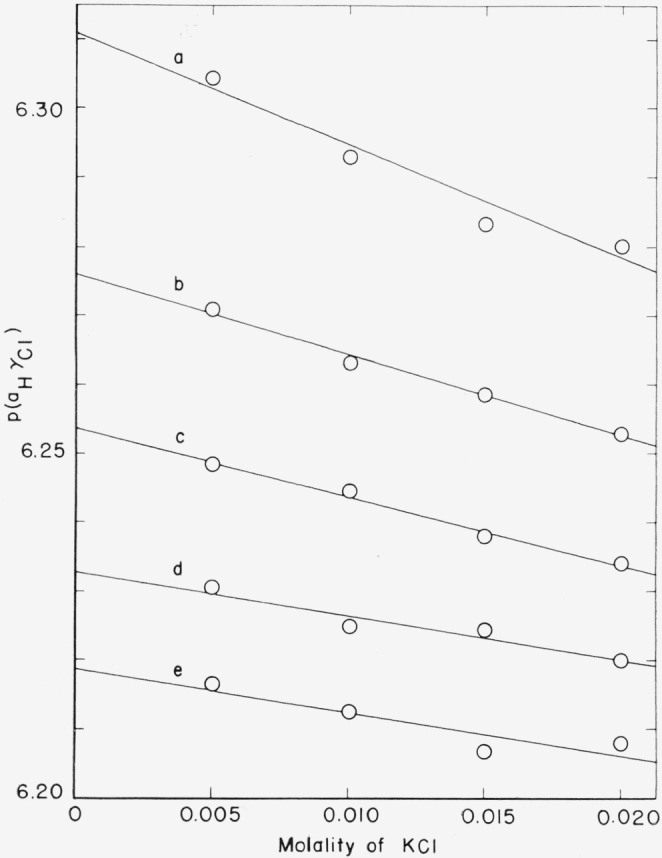
Effect of added potassium chloride on the p(a*_H_*γ*_Cl_*) for equimolal mixtures of potassium dihydrogen phosphate and sodium succinate at 25 °C. Molality of each buffer salt: a, 0.005; b, 0.01; c, 0.015, d, 0.02; e, 0.025.

**Figure 2:**
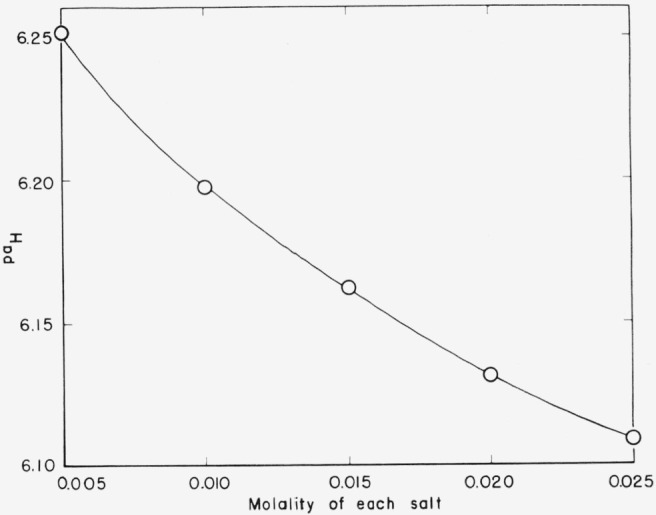
pa*_H_* at 25 °C for equimolal mixtures of potassium dihydrogen phosphate and sodium succinate as a function of the molality of each salt.

The results of the emf measurements and the calculations of *p*(*a*_H_*γ*_Cl_) are summarized in table 1. The average values of *p*(a_H_*γ*_Cl_) corresponding to the four molalities of potassium chloride are plotted as a function of the chloride molality in figure 1. The intercepts at *m*′ = 0, that is *p*(*a*_H_*γ*_Cl_)°, are given in table 2. The intercepts were determined by the method of least squares; the standard deviation of the intercept, *σ_i_*, is given in the third column of the table. The *pa*_H_ values listed in the last column were calculated from *p*(*a*_H_*γ*_Cl_)° together with the conventional definition of the activity coefficient of chloride ion [see eqs (2) and (3)]. The *pa*_H_ of equimolal solutions of potassium dihydrogen phosphate and sodium succinate at 25° is plotted in figure 2 as a function of the molality of each salt.

It is of interest to compare these experimental values of *pa*_H_ with the *pa*_H_ calculated from the dissociation constants for the two acid-base equilibria which fix the acidity of these buffer solutions. The dissociation constant of dihydrogen phosphate ion is given by *pK*_2P_= 7.200 at 25 °C [9], whereas that for succinate ion is expressed by *pK*_2S_=5.636 at the same temperature [8]. From the mass law expressions for the two equilibria,

$$2pa_{H}=pK_{2P}+pK_{2S}-\log\;\frac{m_{P-}m_{S-}}{m_{P=}m_{S=}}-\log\;\frac{\gamma_{P-}}{\gamma_{P=}}-\log\;\frac{\gamma_{S-}}{\gamma_{S=}}$$ (4)

where P and S refer respectively to the phosphate and succinate ions of the indicated charge.

The acid-base reaction taking place when the two buffer salts are mixed in solution may be represented by

$$H_{2}{\text{PO}}_{4}{\;}^{-}+{\text{Suc}}^{=}={\text{HPO}}_{4}{\;}^{=}+{\text{HSuc}}^{-}.$$ (5)

The extent to which this reaction proceeds need not be known, for the concentration term of eq (4) has a value of unity, provided the stoichiometric molalities of potassium dihydrogen phosphate and sodium succinate were equal. Furthermore, the ionic strength (*I*) is equal to 4*m* regardless of the extent to which reaction (5) proceeds.

The last two terms of eq (4) may be estimated as follows. In the course of the investigations which led to the determination of *pK*_2P_ and *pK*_2S_ the quantities log [*γ*_P=_/(*γ*_P=_*γ*_Cl‒_)] and log [*γ*_S=_/(*γ*_S−_*γ*_Cl‒_)] in phosphate buffer solutions and succinate buffer solutions, respectively, were evaluated and found to fit the Hückel equation with ion-size parameters of 4.4 Å and 7.0 Å. If these same parameters are used to calculate the ratios of the activity coefficients of the two phosphate anions and of the two succinate anions in the mixed buffer at 25°, eq (4) then becomes

$$pa_{H}=1/2(pK_{2P}+pK_{2S})-\frac{1.532\sqrt{m}}{1+2.89\sqrt{m}}-\frac{1.532\sqrt{m}}{1+4.60\sqrt{m}}.$$ (6)

The *pa*_H_ values calculated by this equation are compared in table 3 with those derived directly from the emf measurements. The agreement, which is most satisfactory, lends support to the reliability of the experimental data.

The change of *pa*_H_ with temperature can be calculated in a similar fashion. Inspection of eq (4) shows that the effect of temperature changes on the *pa*_H_ of the phosphate-succinate mixture will be a combination of the change of the mean *pK* with temperature and of the temperature effect on the activity coefficients. The heats of dilution of completely dissociated electrolytes are neither large nor very different in the dilute range, and it is justifiable for our purpose to replace d log (*γ*_P=_/*γ*_P−_)/*dT* and *d* log (*γ*_S=_*/γ*_S−_)/*dT* by 3 *d* log *γ_±_*/*dT*, where *γ*± is the mean ionic activity coefficient of one of the common uni-univalent strong electrolytes and *T* is the temperature in °K. From eq (4), one may therefore write

$$\frac{d}{dT}pa_{H}=\frac{1}{2}\frac{d}{dT}\left(pK_{2P}+pK_{2S}\right)-3\frac{d}{dT}\log\;\gamma_{\pm }.$$ (7)

Data in the literature [8, 9] provide the following temperature coefficients:

$$\frac{d\;pK_{2P}}{dT}=0.020912-\frac{2073.0}{T^{2}}$$ (8)

and

$$\frac{d\;pK_{2S}}{dT}=0.019153-\frac{1679.13}{T^{2}}.$$ (9)

Furthermore, it has been shown that *d* log *γ_±_*/*dT* for four common strong electrolytes has the following average values at 25°: −0.00007 deg^−1^ at *I*=0.01, −0.00012 deg^−1^ at *I*=0.04, and −0.0002 deg^−1^ at *I*=0.1.

From these data it may be seen that the contribution of the changes in *pK* to *dpa*_H_/*dT* is −0.00180 deg^−1^ at 20 °C, −0.00107 deg^−1^ at 25 °C, and −0.00038 deg^−1^ at 30 °C. The temperature coefficient of *pa*_H_ at 25 °C for three of the mixed buffers is as follows:

$$\begin{array}{l} \;m\;dpa_{H}/dT\\ 0.005\;-0.00086\;{\text{deg}}^{-1}\\ \;.01\;\;-0.00071\\ \;.025\;-0.0004 \end{array}$$

It is clear that the phosphate-succinate mixture has a lower *pa*_H_-temperature coefficient than either the phosphate buffer or the succinate buffer at room temperature. This is a considerable advantage when the buffer is used in spectrophotometric measurements where the temperature of the absorption cells is not well controlled.

## 5. Application to the Determination of Dissociation Constants

To illustrate the use of these buffer solutions in spectrophotometric work and to obtain a further check on their *p*(*a*_H_*γ*_Cl_)*°* values, determinations of the dissociation constants of two substituted phenols is aqueous solution at 25 °C have been made.

In the spectrophotometric method [1, 10], the dissociation constant *K* of a weak uncharged acid HA,

$$K=\frac{a_{H+}m_{A}-}{m_{\text{HA}}}\frac{\gamma_{A}-}{\gamma_{\text{HA}}},$$ (10)

is determined by measuring the degree of dissociation in a solution of known *p*(*a*_H_*γ*_Cl_)*°* by optical methods. Substitution of *α*, the degree of dissociation, in eq (10) gives

$$pK=p\left(a_{H}\gamma_{\text{Cl}}\right){}^{\circ}-\log\;\frac{\alpha }{1-\alpha }-\log\;\frac{\gamma_{A}-}{\gamma_{\text{Cl}}^{{}^{\circ}}-\gamma_{\text{HA}}}\;.$$ (11)

A small correction is made to allow for the effect of the weak acid on *p*(*a*_H_*γ*_Cl_)° [11].

The succinate-phosphate buffer has been used to measure the dissociation constants of two substituted phenols. Details are given in tables 4 and 5. In the case of 2-nitro-4-cliloroplienol (table 4), the last term of eq (11) has been neglected in arriving at *pK* (corr.) The average of the five *pK* values is 6.45_8_. There is a small but regular upward trend of *pK* with decreasing ionic strength; this may indicate that the last term of eq (11) is not completely negligible, and extrapolation to zero ionic strength, by the method of least squares, gives *pK* 6.46_5_. Either value is in good agreement with that obtained earlier [4].

The value now found for 2,6-dichlorophenol, 6.78_6_, (table 5) is also in good agreement with 6.79_1_, which has been obtained in some unpublished work, using the potassium dihydrogen phospbate-disodium hydrogen phosphate buffer.

## Figures and Tables

**Table 1:** Electromotive force of the cell *Pt; H*_2_(g, *1 atm*), *KH_2_PO_4_*(m), *Na_2_Suc* (m), *KCl* (m′), *AgCl; Ag at 25 °C*. Values of *p*(*a*_H_*γ*_Cl_)

*m*	*m*′							
	0.005		0.010		0.015		0.020	
	*E*	*p*(*a*_H_*γ*_Cl_)	*E*	*p*(*a*_H_*γ*_Cl_)	*E*	*p*(*a*_H_*γ*_Cl_)	*E*	*p*(*a*_H_*γ*_Cl_)
								
0.025	0.72610	6.215	0.70815	6.212	0.69757	6.210	0.68990	6.205
	.72627	6.218	.70830	6.215	.69751	6.209	.69013	6.209
	.72605	6.214	.70806	6.211	.69724	6.204	.68999	6.206
	.72636	6.219	.70810	6.212	.69723	6.204	.69026	6.211
			.70808	6.211				
.020	.72691	6.229	.70896	6.226	.69833	6.223	.69083	6.221
	.72706	6.231	.70879	6.223	.69835	6.223	.69098	6.223
	.72709	6.232	.70872	6.222	.69861	6.227	.69053	6.216
			.70904	6.228				
.015	.72808	6.248	.71005	6.245	.69923	0.238	.69162	6.234
.010	.72945	6.272	.71121	6.264	.70052	6.260	.69273	6.253
	.72936	6.270	.71108	6.262	.70039	6.257	.69272	6.253
.005	.73136	6.304	.71298	6.294	.70191	6.283	.69430	6.279
	.73140	6.304	.71296	6.294	.70193	6.283	.69439	6.281
			.71279	6.291				

**Table 2:** p(a*_H_*γ*_Cl_*)° and pa*_H_* for equimolal solutions of potassium dihydrogen phosphate and sodium succinate at 25 °C

*m*	*p*(*a*_H_*γ*_Cl_)°	*σ_i_*	*pa*_H_
			
0.025	6.219	0.001	6.109
.020	6.233	.001	6.131
.015	6.254	.001	6.162
.010	6.276	.001	6.197
.005	6.311	.002	6.251

**Table 3:** “Observed” and calculated values of pa*_H_* for five aqueous mixtures of *KH*_2_*PO*_4_ and *Na*_2_ *C*_4_*H*_4_*O*_4_ at 25 °C

$m_{{\text{KH}}_{2}{\text{PO}}_{4}}=m_{{\text{Na}}_{2}\text{Suc}}$	*pa*_H_ (observed)	*pa*_H_ (calc., eq(6))
0.025	6.109	6.111
.02	6.131	6.133
.015	6.162	6.160
.01	6.197	6.194
.005	6.251	6.246

**Table 4:** Dissociation constant of 2-nitro-4-chlorophenol in water at 25 °C

Ionic strength^a^	*p*(*a*_H_*γ*_Cl_)°	D^b^	$\log\;\frac{D-D_{1}}{D_{2}-D}$	*pK*	*pK* (corr.)
					
0.10	6.219	0.356	−0.230	6.449	6.448
.08	6.233	.359	−.223	6.456	6.455
.06	6.254	.366	−.206	6.460	6.458
.04	6.276	.374	−.187	6.463	6.460
.02	6.311	.384	−.165	6.476	6.469

Avg. 6.458					
*K*=3.48×10^−7^					

a The molar concentration of 2-nitro-4-chlorophenol was 4.80×10^−5^. The buffer solutions contained equimolar disodium succinate and potassium dihydrogen phosphate.

b The absorption cells were 4 cm in length. *D*_1_, the optical density of the undissociated phenol, measured in 0.01 *M* HCl, was 0.062. *D*_2_, the optical density of the fully ionized phenol, measured in 0.01 *M* NaOH, was 0.855. Measurements were made at 427 m*μ*.

**Table 5:** Dissociation constant of 2,6-dichlorophenol in water at 25 °C

Ionic strength^a^	*p*(*a*_H_*γ*_Cl_)°	D^b^	$\log\;\frac{D-D_{1}}{D_{2}-D}$	*pK*	*pK* (corr.)
					
0.10	6.219	0.365	−0.572	6.791	6.790
.08	6.233	.376	−.556	6.789	6.787
.06	6.254	.389	−.537	6.791	6.789
.04	6.276	.409	−.509	6.785	6.782
.02	6.311	.431	−.478	6.789	6.783

Avg. 6.786					
*K*=1.64×10^−7^					

a The molar concentration of 2,6-dichlorophenol was 8.73×10^−5^. The buffer solutions contained equimolar disodium succinate and potassium dihydrogen phosphate.

b The absorption cells were 4 cm in length. *D*_1_, the optical density of the undissociated phenol, measured in 0.01 *M*HCl, was 0. *D*_2_, the optical density of the fully ionized phenol, measured in 0.01 *M* NaOH, was 1.728. Measurements were made at 300 m*μ*.

## Footnotes

1 Figures in brackets indicate the literature references at the end of this paper.
